# Cellular Impacts of Striatins and the STRIPAK Complex and Their Roles in the Development and Metastasis in Clinical Cancers (Review)

**DOI:** 10.3390/cancers16010076

**Published:** 2023-12-22

**Authors:** Amber Xinyu Li, Tracey A. Martin, Jane Lane, Wen G. Jiang

**Affiliations:** Cardiff China Medical Research Collaborative, School of Medicine, Cardiff University, Cardiff CF14 4XN, UK; lix193@cardiff.ac.uk (A.X.L.); lanej1@cardiff.ac.uk (J.L.); jiangw@cardiff.ac.uk (W.G.J.)

**Keywords:** striatin, STRN3, SG2NA, STRN4, zinedin, STRIPAK, signalling, clinical cancer, prognosis, diagnosis, cancer, Wnt/β-catenin, Hippo signalling, phosphatases, kinases, coiled-coil region, cell proliferation, metastasis, apoptosis, autophagy, cell cycle

## Abstract

**Simple Summary:**

The STRIPAK (striatin-interacting phosphatase and kinase) complex and its central components, striatins (STRNs), have attracted significant interest with regard to the pathological development of diseases over the last decade, particular in the area of cancer research. Beyond their roles in regulating several oncogenic signalling cascades, the clinical relevance of the STRIPAK constituents has intensified research attention towards this supramolecular assembly. However, the complex nature of the complex poses research challenges, and our current understanding provides only a partial view of its functions. As a complex of phosphatases and kinases, its diverse signalling partners further complicate the precise determination of its roles within cancer development. Hence, further research is necessary to unveil and establish its significance as an emerging biomarker. This review consolidates the existing cellular and biological knowledge of STRNs and the STRIPAK members, while also highlighting their demonstrated clinical significance across various cancer types. The current research challenges and future perspectives are also discussed.

**Abstract:**

Striatins (STRNs) are generally considered to be cytoplasmic proteins, with lower expression observed in the nucleus and at cell–cell contact regions. Together with protein phosphatase 2A (PP2A), STRNs form the core region of striatin-interacting phosphatase and kinase (STRIPAK) complexes through the coiled-coil region of STRN proteins, which is crucial for substrate recruitment. Over the past two decades, there has been an increasing amount of research into the biological and cellular functions of STRIPAK members. STRNs and the constituent members of the STRIPAK complex have been found to regulate several cellular functions, such as cell cycle control, cell growth, and motility. Dysregulation of these cellular events is associated with cancer development. Importantly, their roles in cancer cells and clinical cancers are becoming recognised, with several STRIPAK components found to have elevated expression in cancerous tissues compared to healthy tissues. These molecules exhibit significant diagnostic and prognostic value across different cancer types and in metastatic progression. The present review comprehensively summarises and discusses the current knowledge of STRNs and core STRIPAK members, in cancer malignancy, from both cellular and clinical perspectives.

## 1. Introduction

**Figure 1:**
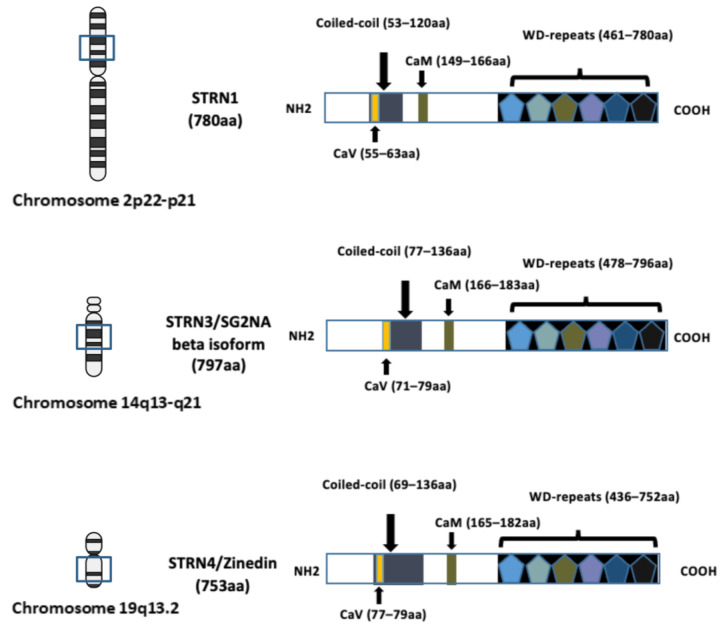
Schematic diagram illustrating the domain structures and chromosome locations of striatin–1 (STRN1), striatin–3 (STRN3) and striatin–4 (STRN4). The structure of each STRN includes four well-recognised domains: the caveolin-binding domain (CaV), the coiled-coil region, the calmodulin-binding domain (CaM) and the WD-repeats domain. The length of the amino acid (aa) sequence is indicated in brackets and the location of corresponding chromosome location is indicated. The arrows in the figure are to indicate location of each domain. Figure created using BioRender.com (agreement number EV266PPC09).

The investigation of the striatin (STRN) family of proteins arose with the identification of striatin-3 (STRN3), also known as SG2NA and PPP2R6B, as a nuclear autoantigen involved in cell cycle regulation [1]. The research on another family member, striatin (STRN1, PPP2R6A), with its name derived from striatium, was concurrently conducted by another research group [2], and was subsequently recognised to belong to the same family as striatin–3 due to shared domain structures and protein–protein interaction domains. Zinedin (striatin–4, STRN4 PPP2R6C and Zin) was later discovered to be homologous to both STRN1 and STRN3 through phylogenetic analysis [3]. The genes encoding STRN–1, STRN–3, and STRN–4 were mapped to chromosomes 2p22-p21, 14q13-q21, and 19q13.2, respectively (Figure 1).

Through affinity purification and mass spectrometry, the STRNs, along with their regulatory protein, protein phosphatase 2A (PP2A), were identified as core constituents of the STRIPAK (striatin-interacting phosphatase and kinase) complex [4]. This complex constitutes an intricate protein network crucial in maintaining cell homeostasis and regulating essential signalling cascades in normal physiological functions. Dysregulation of STRIPAK members has been linked to disease progression, notably in cancer development, a field where their roles have been increasingly elucidated over the past decade.

The general structural composition, architecture, and biological functions of STRIPAK members have been characterised [5,6]. Despite their recognition as emerging cancer biomarkers, a complete understanding of their significance in biological and clinical cancers still needs elucidation. This review will delve into the current understanding of STRNs alongside their associated STRIPAK members in cell signalling events and cellular functions involved in cancer development and progression. More importantly, the clinical relevance of those molecules across cancer types will also be emphasised in this review. 

## 2. Domain Structures of Striatins

The three striatin (STRN) family members share conserved domain structures, including a caveolin-binding region (CaV) at the N-terminal, a coiled-coil domain, a calmodulin-binding region (CaM) and a C-terminal WD40-repeat domain (Figure 1). Although five isoforms of STRN3 have been identified in mice, two of the variants (35 and 38 kDa) lack the C-terminal WD-repeat domain and are primarily membrane-associated [7]. This is distinct from the larger-sized STRN3 variants, which have been found to be cytosolic. A recent study, examining the expression profile of STRN3, confirmed the existence of two larger isoforms (78 and 87 kDas) in human normal mammary tissue and low-staged tumour tissues [8]. 

STRN1 and STRN4 proteins also possess identical domain structures to STRN3, but with slightly different amino acid sequences at the N-terminal region, the region comprising the WD-repeats, and the CaM-binding domains. This variability designated them as proteins responsible for different cellular functions [3]. This conclusion is also supported by Bartoli’s study where, despite the STRN proteins sharing a similar structure, deprivation of STRN1 and the consequent loss in motor function in rats did not appear to be replaced by the expression of STRN3 and STRN4 [9,10]. Indeed, although all three members are expressed in the brain, the three proteins preferentially localise to different areas within the body [3]. With STRN1 enriched in the nervous system and STRN4 in the brain and lung, it is now well-established that STRN3 is ubiquitously expressed in all human tissues, with varying abundance at cytoplasmic, membrane, and nuclear regions of cells. 

Due to the structural composition of STRN proteins, the family members have been shown to directly interact with caveolin and calmodulin, in a calcium-dependent manner, and to be crucial for signal transduction in the nervous and cardiovascular systems. The seven WD40 repeats also serve as a platform for STRNs to interact with other conserved proteins, constituting further downstream signalling events. Of special interest is the α-helices in the coiled-coil region, shown to facilitate dimerisation and oligomerisation between STRNs members. This higher-order association, governed by the coiled-coil region, is fundamental to supporting STRNs in the scaffolding of a range of proteins, some of which are associated with different signalling complexes.

## 3. Architecture of STRIPAK Complexes

**Figure 2:**
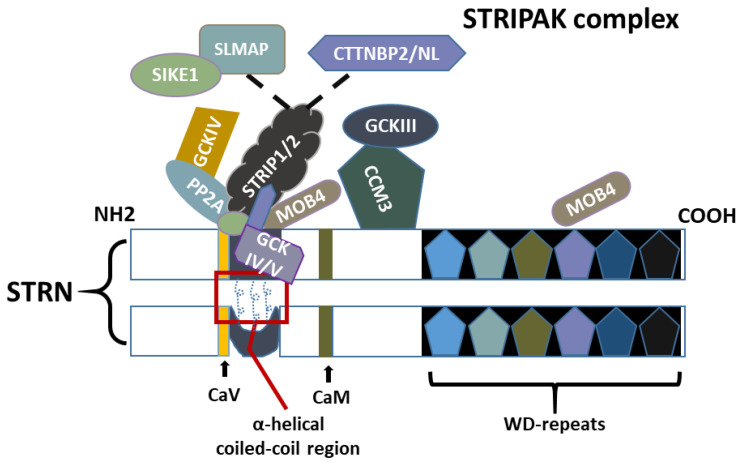
Physical interactions between a constituent member of the striatin-interacting phosphatase and the kinase (STRIPAK) complex. Dimerisation of striatin (STRN) monomers via the coiled-coil region is crucial for substrate recruiting and subcellular targeting. The α-helical chains at the coiled-coil region wind around to form asymmetric homodimers with one chain exhibiting a bend. PP2A (protein phosphatase 2A) interacts with the STRIPAK complex via the coiled-coil region of STRNs. Striatin-interacting molecules STRIP1 and STRIP2 interact directly with the coiled-coil region of STRNs and form mutually exclusive complexes with SIKE1 (suppressor of IKK epsilon-1) and SLMAP (Sarcolemmal membrane-associated protein), or with CTTNBP2/NL (Cortactin binding protein 2 N-terminal like). SIKE1 and CTTNBPs interact with the STRIPAK complex via both the coiled-coil region of STRNs and STRIP1/2. The two MOB4 (monopolar spindle one binder family member 4) at coiled-coil and WD-repeats regions interact with each other in a 3D manner, further bringing PP2A and GCKIII (germinal centre kinase III) members into proximity. CCM3 (Cerebral Cavernous Malformations 3; or PDCD10/TFAR15) also interacts with the STRNs and bridges the interaction between GCKIII and STRN. CaV (Caveolin) and CaM (calmodulin) are known to interact via their respective domains.

STRNs have no intrinsic catalytic activity but serve as scaffolding proteins interconnecting a range of different phosphatases and kinases, forming the striatin-interacting phosphatase and kinase (STRIPAK) complex (Figure 2). With the ongoing advancement of research, more proteins are being discovered to be either tightly or loosely associated with the complex. The specific composition and function of these complexes may vary based on the biological processes being regulated. 

To date, PP2A is the only STRN family-associated protein expressed in all STRIPAK complexes. As shown in Figure 2, other well-known proteins involved in the core complex region include the monopolar spindle one binder family member 4 (MOB4, also known as MOB3 and Phocein), and the germinal centre kinases (GCKs) superfamily of the mammalian sterile20-like kinases (MST1-4 and Ysk1), which bind to STRNs via an adaptor protein called cerebral cavernous malformation protein (CCM3/PDCD10/TFAR15). The misshapen-like kinase 1 (MINK1/MAP4K6), and the striatin interactive proteins STRIP1 and STRIP2 (FAM40A/B) are also contained within the complex. STRIP1/2 can further interact with sarcolemmal membrane-associated protein (SLMAP) and a suppressor of IKK epsilon-1 (SIKE1) or cortactin binding protein 2 (CTTNBP2), in a mutually exclusive manner leading to different forms of the STRIPAK complex for a myriad of cell functions (Figure 2). Additionally, two MINK1 homolog proteins, MAP4K4 and TNIK (MAP4K7 or TRAF2 and NCK-Interacting Kinase), were found to co-precipitate with STRN4 [11], with MAP4K4 found to interact with STRN3 and independently communicate with STRIP1 [12,13]. However, they are not considered core components of the STRIPAK complex due to their event-specific presence and primary roles in distinct downstream signalling pathways. Recently, the Ste20-like kinase (SLK/SLIK), part of the GCKV subfamily, was identified as a new component of the STRIPAK complex, shedding light on the roles of the STRIPAK complex in morphogenesis and cell integrity [14].

### 3.1. PP2A

As mentioned, the coiled-coil domain of STRNs plays a pivotal role in the assembly of the STRIPAK complex. The crystal structure of STRN3 has also been revealed, indicating an asymmetric parallel homodimer configuration, with one α-helical chain at the coiled-coil region exhibiting a bend (Figure 2) [15]. This region facilitates a stable complex formed between STRNs and protein phosphatase 2A (PP2A) with a 2:2 stoichiometry, and this dimerisation is essential for subcellular localisation and substrate recruiting [15]. PP2A is a serine threonine phosphatase composed of a regulatory subunit (B) with a dimer consisting of a catalytic (C) and scaffolding-subunit (A) complex. The A and C subunits represent two isoforms with different redundant functions, whereas the B regulatory subunit is composed of four families (B, B’, B’’, B’’’) of which STRNs members are putative B’’’ regulatory subunits, directing the activity of the PP2A holoenzyme complex in various dephosphorylation-related cellular processes. 

PP2A was initially suggested to be a tumour suppressor, as the inhibition of PP2A by its selective inhibitor, okadaic acid, facilitated tumour growth in mice [16]. Additionally, a tumour antigen SV40 was shown to promote cell transformation by negatively regulating PP2A through displacing the B regulatory subunit [17,18]. As PP2A mutations have been consistently linked with primary tumour formation, numerous studies have speculated on its involvement in oncogenic signalling pathways and cellular functional regulation. However, its exact role in cancer remains incompletely understood. Out of the four regulatory subunits, PP2A trimers containing B and B’ regulatory subunits have been linked to tumour-suppressive activities [19]. However, the presence of the B’’’ regulatory subunit STRNs seems to divert PP2A toward an oncoprotein, by interfering with the Hippo tumour suppressor pathway [20]. Therapies targeting STRNs and associated PP2A dimers might thereby be critical in cancer treatments.

### 3.2. MOB4/MOB3/Phocein

Several proteins that interact with STRNs and PP2A were previously identified and recapitulated in a study, including mammalian Mps one binder (Mob4/Mob3/Phocein), which interacts distinctly with both the coiled-coil and WD40 repeat regions of STRNs (Figure 2) [21,22]. In contrast to PP2A, this interaction is only partially dependent on STRN dimerisation. These studies have also postulated a potential communication between the MOB4 at two ends of the STRNs, bringing PP2A and GCKIII members MST3 into proximity, and promoting the subsequent regulation of MST3 by PP2A [22]. Activated MOB4 has also been reported to form a complex with the other GCKIII family protein, MST4, and the complex is a known regulator of the Hippo-YAP pathway [23].

MOB4 has been shown to interact with all members of the STRNs family, forming complexes at the plasma membrane, cytoplasm and Golgi apparatus and distributing evenly across the regions [21]. Although MOB4 lacks certain sequences that would enable it to become a part of the membrane segment [24], the direct association between STRNs and the plasma membrane facilitates communication between MOB4 and the plasma membrane. Therefore, it is not surprising that both MOB4 and its association with STRNs are implicated in endocytosis and vesicular trafficking, which are critical steps in cancer development [25].

### 3.3. CCM3

The adaptor protein CCM3 (cerebral cavernous malformation), which directly binds to STRNs via its C-terminal region, was initially recognised as an apoptosis-related gene (PDCD10) (Figure 2) [26]. This gene is expressed ubiquitously in humans, encoding a 25 kDa helical protein functioning to coordinate signal transduction between molecules. Its function primarily resides on its N-terminal domain, which can heterodimerise with GCKIII proteins on one side [27] and a focal adhesion targeting homology C-terminal domain on the other side; this is required for interaction with STRNs and other molecules, such as angiogenic vascular-endothelial growth factor (VEGF) and an invadopodium component known as paxillin [28,29]. Apart from several vascular lesions, CCM3 malfunction can also induce abnormal activation of β-catenin and a range of downstream oncogenic signalling pathways [30,31], facilitating endothelial–mesenchymal transition (EMT) involved in several steps of cancer progression. 

### 3.4. GCKs

The GCKs superfamily is part of a broader family of serine and threonine kinases, which includes eight subfamilies (GCKI-GCKVIII). This superfamily conducts kinase activity near their N-terminal domain while also possessing a C-terminal putative regulatory domain. Among these subfamilies, GCKIII (comprising MST3, MST4, and YSK1) and its adaptor protein CCM3, which serves as a bridge facilitating communication between GCKIII and STRNs (Figure 2), are associated with the STRIAPK complex and are putative components of the complex [4]. Although STRNs are likely to bind to GCKIII via CCM3 while in a dimerised form, deletion of the coiled-coil or caveolin-binding domain sequences did not seem to affect the association of GCKIII to STRNs [22]. 

More recently, members of GCKIV, specifically MINK1, MAP4K4, and TNIK, have been shown to directly interact with STRN4, and they are regulated by STRN4 through PP2A [11]. Together they have been proposed to participate in cell cytokinetic abscission. GCKIV was also observed to be part of a complex with STRIP1/2, SLMAP, and CTTNBP2 [11]. As a substrate for STRNs and PP2A, MINK1 is now well-recognised as part of the STRIPAK assembly.

Another GCK family worth mentioning, which shares similar structural characteristics with GCKIII, is the GCKII family. GCKII is considered to be a major component of the Hippo signalling pathway and it functionally serves as a tumour suppressor. The family encompasses MST1 and MST2, in which phosphorylated MST2 has the potential to be bound with the coiled-coil domain of STRN3 [32]. Although they are not obligatory parts of the STRIPAK complex, they have been reported to directly interact and heterodimerise with GCKIII kinases via the C-terminal α helical structure [33]. Indeed, not only are the two families of proteins functionally related to apoptotic processes [34,35], but a recent study has also demonstrated the regulatory function of YSK1 on GCKII members within the Hippo pathway, confirming their involvement in various cancer-related biological processes [36]. 

The last portrayed member of the GCKV family that forms part of the STRIPAK complex is SLIK/SLK [14]. SLK contains a coiled-coil activation loop, facilitating its activation via dimerisation with other kinase domains. The STRIPAK complex has been shown to regulate the subcellular localisation of SLIK. Activated SLIK can mediate moesin activity and it further affects cell integrity and morphogenesis. This is accomplished potentially via STRIP1/2- and GCKs-mediated PP2A dephosphorylation of SLIK. However, this activation is not though the SLIK activation loop. Moesin phosphorylation seemed to require a phosphorylation site of SLIK other than that located on the coiled-coil region of SLIK, as reported by different studies [14,37].

### 3.5. STRIP1/2

Through affinity purification and mass spectrometry, two striatin-interacting molecules, STRIP1 and STRIP2, were found to be contained within the same stable complex as PP2A, via binding with the coiled-coil domain of STRN members via their N-terminal [4,29,32]. It is now well-established that the STRNs-associated STRIP1/2 may form mutually exclusive complexes with SLMAP and SIKE or with CTTNBP2 (Figure 2). STRIP1 and STRIP2 share homology, with approximately 60% identity at the amino acid level. They perform slightly different roles, as cells with reduced expression of STRIP1 versus STRIP2 were shown to exhibit distinct morphology phenotypes and actin cytoskeleton alternations, further affecting their contractility, cell adhesion and migration [38]. Although the two molecules do not physically bind to CCM3 and GCKIII, patterns of them being co-expressed in vitro were proposed in different studies [4,38]. STRIP1 also functions as a negative regulator for GCKIII members in cancer cells [39]. Additionally, STRIP1/2 have been shown to recruit GCKII and directly bind to activated kinases in a phosphorylation-dependent manner, allowing modulation of Hippo signalling by the STRIPAK complex [32]. 

### 3.6. SLMAP/TRAF3IP3

SLMAP is a tail-anchored membrane protein with a C-terminal transmembrane domain inserted into the plasma membrane. Its domain sequences not only allow it to form a heterodimer with SIKE1 but also enable direct interactions with the C-terminal regions of STRNs at the nuclear membrane (Figure 2) [29,40]. Additionally, SLMAP contains an N-terminal forkhead-associated (FHA) domain that is crucial for recognising phosphorylated peptides. The relationship between SLMAP and STRN3 was further elucidated in pulldown assays, which suggested the requirement of SIKE1 for this interaction [32]. Similar to STRIP1/2, SLMAP directs the STRIPAK complex to phosphorylated MST1/2 and binds to activated MSTs via its FHA domain, whereas its interaction with GCKIV family proteins was rarely observed [41,42]. Beyond their inhibitory roles in Hippo activation [43], the expression of SLMAP and MOB4 has been found to be positively associated with activated moesin levels, linking them to multiple morphogenetic processes [14].

Unlike SLMAP, TRAF3-interacting protein 3 (TRAF3IP3) has an uncharacterised N-terminal domain structure and shares only 22% amino acid identity with its paralog, SLMAP. However, both molecules have been shown to be immunoprecipitated with SIKE1 and are expressed in the STRIPAK complex in a mutually exclusive manner with CTTNBP2 (Figure 2). TRAF3IP3 is known for its function in antiviral infection and T-cell immunity [44]. However, as a STRIPAK partner and due to its involvement in various signalling pathways, a growing body of research and evidence has suggested its roles in cancer progression.

### 3.7. SIKE1/FGFR1OP2

As mentioned, SIKE1 forms a heterodimer with SLMAP in the STRIPAK complex, achieved via their first coiled-coil domains (Figure 2). SIKE1’s structure contains three coiled-coil regions, with the second coiled-coil domain (residues 72–121) forming a complex with STRN3. This interaction occurs within the region spanning residues 64–190 of STRN3, primarily composed of the coiled-coil α-helix structures [32]. Although the depletion of SIKE1 and several other STRIPAK members, namely SLMAP, STRIP1, and STRNs, significantly promote the activation of MST2, all except SIKE1 were found to directly interact with the kinase [32].

FGFR1OP2 is a paralog of SIKE1 and part of the STRIPAK complex. Although the expression of FGFR1OP2 has been implicated in wound closure, its role in cancer and involvement in the STRIPAK complex have not been fully elucidated. However, recent predictions suggest that this protein may bind with CCM3, a known oncogene involved in the formation of secondary cancers [45].

### 3.8. CTTNBP2/CTTNBP2NL

CTTNBP2, along with its paralog CTTNBP2 N-terminal like protein (CTTNBP2NL), has been reported to be associated with PP2A in HEK293 cells [4]. Although they exhibit high sequence similarity and were suggested to be the same type of molecule, they show distinct distributions and functions, at least in neurones. Specifically, CTTNBP2 but not CTTNBP2NL interacts with STRNs via the coiled-coil regions of both molecules [46]. However, a recent study has identified CTTNBP2NL and STRN4 as directly interacting with molecules MINK1 and TNIK [42]. Together with the results from Hyodo’s study [11], it is highly likely that CTTNBP2NL shares redundant roles with MINK1 and TNIK in related biological processes. 

## 4. Cell Signalling Events Involved with STRNs and the STRIPAK Complex

### 4.1. Hippo Signalling Pathway

As mentioned, MST1/2 are downstream effectors of the STRIPAK complex and are crucial regulators in the Hippo signalling pathway, known to exhibit a tumour-suppressive role by regulating cell growth, proliferation, and apoptosis. However, recent studies have challenged the traditional understanding of Hippo signalling in cancer. Owing to its tissue-type-specific roles in tumourigenesis, its activation has been proposed to promote tumour growth in colorectal and ER+ breast cancer [47,48].

In general, the activated MAP4Ks could act in parallel with MST1/2 to phosphorylate large tumour suppressor kinase (LATS1/2). LATS1/2 then cooperates with a scaffolding protein MOB1, to phosphorylate and prevent translocation of Yes-associated protein 1 (YAP) and WW-domain-containing transcription regulator 1 (TAZ) into the nucleus, to bind with the transcriptional enhanced associated domain (TEAD) proteins. This disrupted association results in transcriptional silencing. Therefore, dysregulation of Hippo cascade-induced hyperactivation of YAP/TAZ has been shown to drive cancer development and progression.

**Figure 3:**
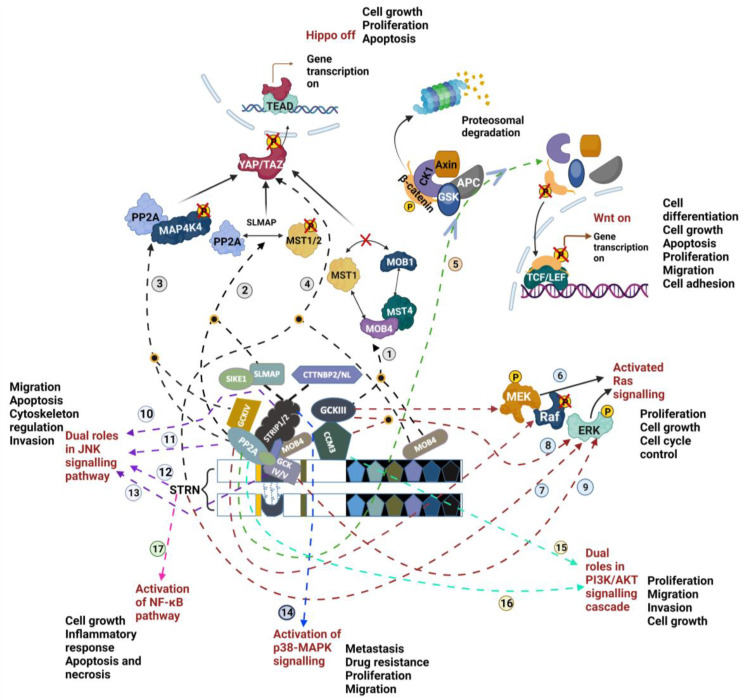
Mechanisms of STRIPAK involvement in different signalling pathways. The lines arise from the STRIPAK members and lead to Hippo (black dotted pathway 1–4), Wnt (green dotted pathway 5), Ras (red dotted pathway 6–9), JNK (purple dotted pathway 10–13), p38/MAPK (blue dotted pathway 14), PI3K/AKT (blue-green dotted pathway 15–16) and NF-κB (pink dotted pathway 17) signalling pathways. The potential implications of STRIPAK complex-induced signalling cascade alterations for changing cell behaviour are also listed next to the corresponding signalling cascade. Details of each modulation pathway are described in the main text. A yellow circle filled with black indicates the convergence of two pathway lines. Figure created using BioRender.com (agreement number KW267CSUF2). YAP—Yes-associated protein 1; TAZ—WW-domain-containing transcription regulator 1; TEAD—transcriptional enhanced associated domain; TCF/LEF—T-cell factor/lymphoid enhancer factor; P—Phosphate; GSK3—glycogen synthase kinase 3; CK1—casein kinase 1; APC—adenomatous polysis coli).

Although the exact role of STRIPAK in Hippo remains undetermined, one of the STRIPAK members, MOB4, has been speculated to be involved in Hippo signalling, through its phosphorylation-dependent binding with MST4 [23]. The MOB4-MST4 complex has been suggested to antagonise the interaction between MOB1 and MST1 and to abolish the downstream phosphorylation of LATS1/2 (Figure 3 pathway 1). The idea postulated is that the two complexes have a conserved structure, allowing the two MOBs to pair up with either of the MSTs to hinder their original function. However, their amino acid sequences differ in detail, with some of the key residues mediating the MOB1-LATS interaction lacking in MOB4. This has led to persistent YAP/TAZ activation, which has been shown to drive cell growth and migration in pancreatic cells [49]. Additionally, as mentioned earlier, in the role of SLMAP directing PP2A to MST1/2, the MST1/2 scaffolding protein Salvador homolog 1 (SAV1) has been shown to antagonise the recruitment, restricting the dephosphorylation of MST1/2 by PP2A (Figure 3 pathway 2) [43]. The STRIPAK complex-meditated dephosphorylation of MAP4K4 by PP2A may also interfere with the downstream activation of LAST1/2 and MOB1 [50] (Figure 3 pathway 3).

However, the regulation of kinases and phosphatases normally functions in a reciprocal and compensated manner, to maintain basal cellular signalling and processes. Although both STRIP1/2 and SLMAP have been shown to mediate the MSTs’ dephosphorylation, investigating the regulation of the STRIPAK complex is crucial for understanding its broader function in cellular regulation. In this context, the STRIPAK complex has been demonstrated to play an essential role in both activating and inhibiting the Hippo cascade by integrating its upstream signals. Chen et al., have proposed a RhoA-rhophilin-NF2/Kibra-STRIPAK signalling axis in the regulation of the Hippo pathway [13]. 

### 4.2. Wnt/β-Catenin

Activation of the Hippo cascade leads to suppressed gene transcription, whereas the canonical Wnt/β-catenin pathway is often found to be aberrant, inducing activation of numerous oncogenic genes. Although PP2A is a known modulator of this signalling cascade [51], other STRIPAK members, namely STRN1 and MOB4, have recently been shown to affect differentiation and cell size by interacting with Wnt proteins [52]. The STRIPAK complex has been proposed to suppress Wnt signalling. As illustrated in Figure 3, this signalling cascade is initiated by the incorporation of Wnt proteins into the transmembrane low-density lipoprotein (LRP) and Frizzled (FZD) receptors. The activated receptors recruit the cytoplasmic destruction complex, including glycogen synthase kinase 3 (GSK3), casein kinase 1 (CK1), and axin and adenomatous polysis coli (APC) to the plasma membrane, to prevent phosphorylation of CK1 and GSK3, which can initiate degradation of cytoplasmic β-catenin. Stabilised β-catenin can then translocate into the nucleus, bind with T-cell factor/lymphoid enhancer factor (TCF/LEF) and ultimately induce uncontrolled cell proliferation and migration. 

Since most of the components within this signalling cascade can be modulated by phosphorylation, it is not surprising that PP2A is involved in this process (Figure 3 pathway 5). Indeed, it has been demonstrated that instead of dephosphorylating β-catenin, PP2A can negatively regulate Wnt signalling through signalling with GSK3β and APC [53]. When there is over-activation of Wnt/β-catenin signalling, PP2A is likely to compensate for this effect via the dephosphorylation of axin, which in turn, facilitates β-catenin accumulation. Additionally, APC, this core Wnt/β-catenin signalling component, has recently been demonstrated to interact with STRNs at tight junctions to maintain normal cell–cell adhesion [54].

### 4.3. MAPKs

Mitogen-activated protein kinase (MAPKs) cascades are ubiquitous signal transduction pathways, involved in regulating gene expression and cellular functions at all levels. To date, at least three MAPK signalling pathways have been characterised, one of which is known as the Ras-Raf-MEK-ERK (Ras) pathway. Its sustained activation, triggered by the activation of epidermal growth factor receptor (EGFR), has been implicated in cell proliferation and cancer development. The STRIPAK complex, functioning downstream of EGFR and acting as a positive regulator of the Ras signalling cascade, has been proposed in both *Drosophila* and mammalian cells [5]. This regulation of Ras occurs through the PP2AC and GCKIII-mediated basal phosphorylation of ERK, facilitating the activation of target genes (Figure 3 pathway 6). MEK contains serine/threonine phosphorylation sites and can be activated by GCKIII members [55], whereas Raf is activated upon its dephosphorylation by PP2A [56].

Many MAPKs, such as MINK1 and MAP4K4, are considered either a part of the STRIPAK complex or direct substrates of it. Thus, the complex’s contribution to the Ras cascade has been proposed in various studies. STRNs homolog, now known as *connector of kinase to AP-1* (Cka) is found in *Drosophila melanogaster* and participates in a range of different signal transduction pathways.

Knocking down of STRNs and Cka attenuated ERK phosphorylation and significantly reduced cell proliferation (Figure 3 pathway 7) [57]. Modulation of Ras signalling by CCM3 was also reported to facilitate tumour transformation of cells, which were shown to be mediated by MST4 (Figure 3 pathway 8) [55]. While MINK1 was demonstrated to be downstream of the Ras cascade, mediating cell cycle arrest in ovarian epithelial cells [58], its homolog MAP4K4 was found to be overactivated in lung cancer, stimulating growth and migration of cells by activating ERK in the Ras signalling cascade (Figure 3 pathway 9).

The STRIPAK complex has been linked to another MAPK signalling pathway, known as the Jun N-terminal kinase (JNK) signalling pathway. In *Drosophila*, Cka has been shown to facilitate the morphogenetic movement of epithelial cells and apoptosis in wing imaginal discs, by positively regulating the JNK cascade [59]. However, the roles of other STRIPAK members in the JNK pathway have yet to be fully elucidated, as the definite binding partner of Cka, the phosphatase PP2A, is known to negatively regulate JNK (Figure 3 pathway 11). Although Cka can activate JNK-downstream transcription factors in the nucleus, contributing to various cellular responses, it has also been reported to have inhibitory roles in JNK signalling (Figure 3 pathway 12) [60]. Additionally, STRIP1/2 has been shown to suppress JNK signalling and is required to prevent JNK-mediated apoptosis (Figure 3 pathway 10) [61]. MAP4K4 is known to activate JNK and has been implicated in cell motility and cytoskeleton regulation in cancer invasion and dissemination (Figure 3 pathway 13) [62].

The last MAPK pathway is referred to as the p38-MAPK signalling cascade. Its activation is considered an oncogenic process that contributes to both metastasis and resistance to chemotherapy. Although its connection with the STRIPAK complex in cancer remains unclear, the deactivation of p38-MAPK upon inhibition of STRIP2 sheds light on the regulation of STRIPAK in this signalling cascade (Figure 3 pathway 14). This deactivation subsequently hindered the proliferation and migration of mouse aortic smooth muscle cells [63]. Further research is needed to uncover the precise roles of the STRIPAK complex in the MAPKs signalling cascade.

### 4.4. PIK3/AKT

Cell functions are governed by a variety of signalling pathways, some of which collaborate with each other or function in a compensatory manner to maintain cellular balance. The interplay between PI3K/AKT and the MAPK-Ras signalling pathways has long been established. Beyond the previously mentioned contributions of STRIPAK members to RAS signalling, recent research has suggested that CCM3 may influence the phosphorylation and activation of AKT and ERK (Figure 3 pathway 15). This consequently prompts oncogenic features of pituitary adenoma cells, including EMT, increased cell proliferation, migration, and invasion [64].

The STRIPAK members have also been proposed to orchestrate the Hippo signalling and PI3K/AKT cascades in *Drosophila*. In this context, PP2A mediates cell growth arrest by inhibiting PI3K/AKT signalling (Figure 3 pathway 16), whereas Cka and MOB4, core components of the STRIPAK complex, are essential for PP2A’s association with the Hippo signalling pathway, leading to cell reactivation (Figure 3 pathway 4) [65].

Looking at how a downstream effector can mediate the activities of an upstream regulator in a negative feedback loop, to maintain proper cell homeostasis, research has demonstrated a regulatory role of ERKs for STRNs in addition to being a downstream signalling pathway of the STRIPAK complex [66,67]. Additionally, an upstream regulatory mechanism of STRNs, by E2 via the PI3K/AKT axis, has also been proposed by the same research group [67]. Their study suggests that E2-mediated upregulation of STRNs plays a role in cell migration, but it appears to be tissue-dependent.

### 4.5. NF-κB Signalling

Despite the aforementioned signalling pathways, new insights have focused on the regulatory properties of PP2A in the nuclear factor kappa light chain enhancer of activated B cells (NF-κB) pathway, mediated by STRNs (Figure 3 pathway 17). STRNs were shown to phosphorylate cascade member IKKβ, subsequently inducing translocation of NF-κB to the nucleus, where it leads to the transcription of different genes [68]. This cascade has been shown to participate in cell growth and mediate inflammatory responses, which are important in cancer development. NF-κB also stimulates anti-apoptosis and necrosis machinery and is in close association with ER stress and cell homeostasis [69].

## 5. Cellular Function of STRNs and the STRIPAK Complex

The STRNs have been found to possess evolutionarily conserved functions and have been studied across different eukaryotic species, through the examination of their homologs. Serving as a cornerstone of the supramolecular STRIPAK complex, STRNs govern the regulation of various phosphatases and kinases, and have thereby been suggested to be extensively involved in a wide range of biological and cellular processes.

### 5.1. Cell Cycle

STRN3 is a well-recognised protein known to have elevated expression during the S and G2 phases of the cell cycle. Upregulation of STRN3 is associated with the extension of the G2 phase duration, whereas downregulation of STRN3 induces cell cycle arrest at G0/G1 [66]. By manipulating the expression of individual STRIPAK members, a recent study has provided further insight into the role of STRIP1 in regulating the G1 phase of the cell cycle [39]. This regulation occurs through GCKIII-mediated enhancement of the expression of cyclin-dependent kinase (CDK) inhibitors p21 and p27, following STRIP1 depletion. Furthermore, STRNs have been implicated in modulating microtubule dynamics, which plays a fundamental role in key cell cycle events such as chromosome segregation, spindle assembly, and cytokinesis [70]. 

### 5.2. Cytoskeleton Remodelling

The cell cytoskeleton consists of microtubules, actin, and intermediate filaments, forming a dynamic and extremely complex network that controls not only cell integrity, architecture, and motility but also signals to cells in response to external stimuli. The implication of the STRIPAK complex in cytoskeleton remodelling is often associated with the ezrin, radixin and moesin (ERM) family proteins, a group of proteins linking the cytoskeleton and cell membrane that are key modulators in cell morphology/migration and tissue morphogenesis. Inhibition of PP2A in the STRIPAK complex has been shown to affect the subcellular localisation of SLIK and downregulate ERM in *Drosophila*, resulting in morphological defects of cells during mitosis [14].

During endothelial cell migration, lamellipodia formation and membrane retraction have been demonstrated to be, in part, due to MAP4K4-regulated ERM phosphorylation, in response to growth factor stimulation [71,72]. In addition to STRIPAK association with the ERM proteins, STRIP1 and STRIP2 have been identified as regulators of the cytoskeleton [73]. However, their depletion induces distinct phenotypes of cells, indicating differences in their signalling partners during cell spreading. Although several lines of evidence have shown the involvement of the STRIPAK complex in cytoskeletal organisation, the study of individual STRIPAK members in this area is lacking. Until recently, STRN1 has been found to co-localise with microtubules in HEK293T cells, and its downregulation resulted in microtubule depolymerisation [70]. However, the exact signalling events involved in this process need further elucidation.

### 5.3. Apoptosis and Autophagy

Apoptosis is recognised as programmed cell death. Although its dysregulation can induce tumour progression and angiogenesis in cancer, it is also a leading cause of resistance to chemotherapies. All three members of the STRNs family have been implicated in this manner, with decreased expression associated with increased cell susceptibility to apoptosis, under cell stress or cancer therapies [42,59]. However, in contrast to traditional results, a recent study offers a new perspective that STRNs can regulate cell apoptosis in T-cells when in complex with the scaffolding subunit of PP2A (PP2AA). The regulation is possibly via NF-κB signalling [15]. During cancer development, ER stress can enhance cell aggressiveness by promoting NF-κB mediated anti-apoptosis; and this activity has further been found to be facilitated by the STRN3–PP2A complex [69]. Beyond the core region, other STRIPAK components also play roles in apoptosis, via influencing various signalling cascades. For example, MAPKs were found to inhibit Hippo signalling via MST1/2 [42], whereas CCM3 regulated MST4-dependent ERM phosphorylation as a protective mechanism for cells to survive oxidative stress-induced apoptosis [74].

Autophagy demonstrates controversial roles in tumour formation, suppressing tumour initiation by eliminating damaged components while potentially fostering cancer development through increasing cell susceptibility to stress-induced death. A recent study has revealed interaction between STRN1 and ULK1 (Unc-51 Like Autophagy Activating Kinase 1) in a positive-feedback manner via PP2A, promoting cell autophagy [75]. The implication of STRIPAK in autophagy within muscle tissues has additionally been demonstrate in cancer research [76,77]. Although MAP4K2, a GCKI family member, is not considered a STRIPAK member, the depletion of STRN4 induces hyperphosphorylation and activation of MAP4K2, suggesting a crucial regulatory role of STRIPAK upstream to MAP4K2. This regulation has been proposed to be involved in autophagy during cellular stress [77]. 

### 5.4. Cell Adhesion and Migration

With sufficient evidence demonstrating the contribution of STRIAPK members in cytoskeleton regulation, it is not surprising that this complex exerts significant roles in cell adhesion and motility. Consequently, the complex’s members may offer novel insights into understanding cancer development and progression. Recent findings indicate that STRN1 colocalizes with APC, a regulatory protein of tumour-suppressive β-catenin, at cell contact areas, helping to maintain correct cell–cell adhesion [54]. STRN1 was not found to colocalize with E-cadherin, an adherens junction marker. However, its depletion reduces cell adhesion through mechanisms involving E-cadherin. Furthermore, alterations in E-cadherin affect the subcellular localisation of STRN1, subsequently influencing cell polarization and migration [54].

PP2A has been shown to negatively regulate tight junction assembly through interactions with several junctional proteins such as occludin [78]. This regulation may be mediated by different regulatory subunits of PP2A, as APC has been found to direct PP2A activity by interacting with several distinct regulatory subunits of PP2A [54]. Nevertheless, the contribution of the STRIPAK complex, in addition to STRN-APC or individual STRIPAK member-mediated cell behaviour changes, remains to be addressed. 

### 5.5. Cell Proliferation

STRIPAK complex components are involved in cell proliferation, via the regulation of several signalling pathways. Knocking down of STRN3, SIKE1, SLMAP, and STRIP1 was associated with increased MST2 activation, which, in turn, inhibits YAP translocation and oncogenic gene transcription in the gastric HGC27 cell line [79]. Additionally, the STRN3/PP2A complex was shown to deactivate MAP4K4 in Hippo signalling, promoting cell growth and proliferation in brain tumour cells [12]. With the involvement of STRIPAK in cell cycle control, the breast cancer cell line MDA-MB-231 exhibited cell cycle arrest and suppressed cell proliferation and growth upon depletion of STRIP1. This regulation was shown to involve tumour-suppressive CDK inhibitors p21 and p27 [39]. Furthermore, MOB4 is also a crucial STRIPAK component that controls cell proliferation by involvement in STRIPAK assembly and regulation of Hippo signalling. Downregulation of STRN1 has been shown to destabilise microtubules and inhibit the proliferation of HEK293 cells [70].

### 5.6. DNA Damage and Repair

As key machinery involved in the loss of the tumour-suppressive Hippo signalling pathway, STRIPAK complex-mediated MST1/2 inactivation of Hippo has been shown to facilitate DNA double-stranded break (DSB), inducing resistance to drug therapies [80]. Interestingly, although STRIP1 depletion has a tumour-suppressive role in breast cancer proliferation via communication with P21 and P27, it showed a contradictory characteristic upon the administration of chemotherapies to cells [39]. The cells’ fate, after non-lethal doses of chemotherapy, is dependent on the STRIP1-mediated P21/P27 expression level prior to and during the drug treatment. Inhibition of PP2A was additionally investigated in a recent study as a way of sensitising cancer cells to radio- and chemotherapies [81]. 

### 5.7. Immune Regulation

More and more research has recently appreciated the role of STRIPAK members in immune regulation in cancer. STRN4 and PP2A exhibited immunosuppressive features that were shown to limit stimulator of interferon gene (STING)-mediated antitumour immune responses, via modulating the Hippo signalling pathway [82]. Similarly, reduced expression of STRIP2 was associated with a higher level of immune response in lung adenocarcinoma [83]. Additionally, MAP4K4 has also been shown to regulate inflammation-related signalling cascades such as the NF-κB pathway [84]. It has been established that cancer cells can mimic ligands of immune checkpoints, as a way to escape immune surveillance. The immunotherapy drugs function as inhibitors, blocking this false interaction. Recently, PP2A has emerged as a key immune-checkpoint regulator, but again, its substrate specificity and involvement in downstream signalling is varied, depending on its regulatory subunits [85].

### 5.8. Therapeutic Drug Responses

Despite the direct relationship between the alteration of many aforementioned cell functions and drug responses, the exact role of the STRIPAK complex in both cellular and patients’ responses to chemotherapies remains inconclusive and largely undetermined. This uncertainty may stem from signalling variability across different cells and tissues, as well as cancer types.

Although a lower level of STRIP1 was shown to supress breast cancer cell proliferation via interference with cell cycle regulation, decreased STRIP1 exhibited tumour-promoting effects when cells were treated with a non-lethal dose of doxorubicin or cisplatin [39]. STRIPAK-mediated deactivating of MST1/2 in Hippo signalling also endows cells with resistance to chemo-, radiotherapies, and PARPi (poly ADP ribose polymerase inhibitor), an inhibitor to modulate DNA repair [80]. Furthermore, silencing of STRN4 was shown to sensitise pancreatic cancer cells to gemcitabine [86]. Clinically, breast cancer patients who expressed higher level of STRN3, CCM3, PPP2CA, and PPP2CB were shown to be more sensitive to chemotherapies. However, the involvement of STRN3 in breast cancer patients’ drug responses was also reported to be hormone-receptor-status dependent [87].

### 5.9. Other Cellular Functions of the STRIPAK Complex

STRN1 has recently been identified as a regulator of ER homeostasis, as its depletion impairs the adaptive unfolded protein response (UPR) under ER stress, resulting in cell death [88]. SLMAP has also been found to localise in the ER, yet its role in ER homeostasis has not been clarified [89]. The same study has proposed the presence of SLMAP in mitochondria and the nuclear envelope, looking at the roles of STRNs, STRIP, and SLMAP in linking STRIPAK to those different locations. Indeed, several studies have established the role of STRIPAK in nuclear import of MAPKs in a dependent manner in fungi and its role in cell metabolism [40,90]. Furthermore, STRIPAK members have been demonstrated to regulate angiogenesis processes [38], which is a critical step involved in tumour growth and cancer progression. 

## 6. STRNs and STRIPAK in Cancer, Cancer Metastasis, Clinical Manifestation and Patients’ Survival

### 6.1. Overall Involvement in Cancer

The discovery of STRIPAK complex has shed light on the potential roles of STRNs in cancer. These initial observations have also sparked significant interest in investigating members of the STRIPAK complex in specific human cancer types. As summarised in Table 1, recent research has established clinical links between the STRIPAK members and the progression of various cancer types, as well as clinical outcomes of patients. However, the coverage of cancer types and the number of STRIPAK members investigated in these studies was limited, and some research also presented contrasting perspectives. 

As the cornerstone of the STRIPAK complex, the STRNs may direct STRIPAK complex members through different signalling pathways, thereby exhibiting dual regulatory properties during cancer progression. Therefore, more research focusing on the central scaffolding proteins, STRNs, is required. Lab-based research is necessary to elucidate the morphogenic roles of STRNs in different steps of cancer development and their tissue-specific properties. The following will summarise the key findings of the core STRIPAK members regarding their involvement in the development of specific cancer types.

### 6.2. Breast Cancer

In breast cancer, STRN3 was found to have increased expression in breast cancer tumour tissues compared with adjacent normal tissues, at both the transcript and protein levels [8,87]. Our previous findings also indicate that STRN3 expression is associated with chemo-drug response in patients with positive oestrogen receptor status (ER+) [82]. Indeed, its homolog STRN1 is known to direct ERα trafficking and initiate several signalling pathways in endothelial cells. Breast cancer development is frequently driven by hormonal fluctuations, making the regulation of its receptor crucial for tackling disease progression. Together with research on the regulatory roles of ERα in chemoresistance in breast cancer cells [122], our previous results further establish a receptor-dependent role of STRNs in breast cancer patients’ drug responses [87]. The study has also found that the expression pattern of a subset of the STRIPAK complex members forms an independent prognostic indicator for the overall survival and the disease-free survival of the patients. 

Additionally, the activation of MST3/4, which can be antagonised by the presence of STRNs and STRIP1 in breast cancer cells, was shown to facilitate cell migration by activating ERM proteins and inhibiting myosin light chain dephosphorylation [99]. However further research is required as MST3/4 were not the only molecules regulating the change in the cytoskeleton, and the ultimate change in cell morphology is often coordinated by different signalling pathways. 

### 6.3. Gastric Cancer

It has been shown that the STRIPAK complex holds promise for inhibiting Hippo-associated tumour-suppressive activity in gastric cancer [80]. This is regulated by STRN3-mediated dephosphorylation of MST1/2 [32]. The loss of Hippo signalling subsequently stimulates the repair of DNA double-stranded breaks (DBSs) and endows gastric cancer cells with elevated resistance to radio-and-drug therapies [80]. According to Tang [32], strikingly increased STRN3 protein expression was observed in the gastric cancer cell line HGC27 compared with the normal gastric epithelium cell GES-1. Its expression was positively correlated with YAP activation and poor prognosis of gastric cancer patients. Inhibition of STRN3 reduced the formation of HGC27 cells colonies, and a similar inhibitory effect was additionally observed in vivo in xenograft mouse models, with reduced tumour size following STRN3 inhibition [32]. Recently, targeting STRN4 by microRNA-6165, resulting in reduced STRN4 production, was also shown to suppress gastric cancer cell migration and invasion [95]. Those results further provide insight into the diagnostic and prognostic values of STRNs and the STRIPAK complex in gastric cancer.

### 6.4. Pancreatic Cancer

In limited research on STRIPAK members in pancreatic cancer, the expression of STRN4 was found to be associated with reduced cell growth and spreading both in vitro and in vivo in mice [86]. Knocking down STRN4 sensitised pancreatic cancer cells to gemcitabine. CCM3 has additionally been demonstrated to express higher levels in tumours than in normal pancreatic tissues. Higher expression further indicated shorter overall survival of pancreatic cancer patients [45].

Information regarding the involvement of the STRIPAK complex in Hippo signalling has shown that the interaction between MST4 and MOB4 facilitates disassembly of the MST1MOB1 complex and, in turn, inhibits YAP phosphorylation in pancreatic cancer. This has resulted in increased cell proliferation and aggressiveness [23].

Recently, several STRIPAK complex members were implicated in EMT, a collective process crucial to driving cancer progression and resistance to drug therapies. By analysing multiple online datasets and conducting in vitro work on pancreatic cancer cells, STRN1 and STRN3 were found to have upregulated expression in pancreatic tumour tissues and were associated with poor patient survival, in contrast to PPP2R1B, a scaffolding subunit of PP2A whose downregulation was shown to promote EMT [109]. In vitro RNA interference had also been applied to inhibit the activity of MAP4K4, which is considered as oncoprotein in pancreatic adenocarcinoma [84,123]. 

Since the STRIPAK complex integrates the expression of various phosphatases and kinases exhibiting different roles in disease progression, a well-balanced expression between those molecules is significant for maintaining a healthy pancreas.

### 6.5. Oesophageal Cancer

Apart from being a novel poor-prognosis indicator for oesophageal carcinoma patients [91], cells with reduced STRN4 expression behaved less aggressively compared to the control cells, as reflected by reduced proliferation, invasion and migration [86]. An upstream oncogenic regulator of STRN3, miR-885-5p, was recently identified and found to facilitate malignant behaviour in oesophageal cancer cells via regulating the STRN3-mediated Hippo cascade [92]. Integrated expression of STRIPAK genes was also assessed in an oesophageal cancer patient cohort. Together, dysregulation of STRIPAK genes exhibited significant diagnostic and prognostic values in this cancer type [113].

### 6.6. Thyroid Cancer

Although patients with thyroid cancer generally showed better clinical outcomes compared to other cancer types, a subset of thyroid tumours is highly aggressive and is associated with an increased mortality rate in patients. A recent study has identified *STRN1-ALK* (anaplastic lymphoma kinase) gene fusion as a novel marker in thyroid cancer, with the fused genes showing a higher prevalence in tumours exhibiting increased aggressiveness [124]. The same gene fusion was also observed in non-small cell lung cancer (NSCLC) and renal cell carcinoma [125,126], the NSCLC patients were shown to benefit from ALK inhibitors [126].

It has been shown that the dimerisation of STRN1 and ALK is mediated by the coiled-coil region of STRN1 [124], an area that also facilitates interaction with PP2A and mediates activation of Hippo. Increased expression and activation of YAP1 have been reported in thyroid cancer cell lines and tissues [127], making the coiled-coil region of STRNs a potential therapeutic target in cancers with STRN-ALK fusion.

### 6.7. Lung Cancer

Tracing back to the discovery of STRN3, it was identified as a nuclear autoantigen in the serum of lung and bladder cancer patients, revealing elevated expression during the S and G2 phases of the cell cycle [128]. Cancer cells often feature uncontrolled proliferation, making it highly likely that STRN3 is dysregulated during the process of cancer development. Although the clinical significance of STRNs in lung cancer has not been fully established, associated STRIPAK complex members have been reported to have roles in lung cancer development and prognosis. 

In addition to STRN-ALK fusion, *ALK* gene rearrangements may also lead to novel fusion of *ALK* and *SLMAP* genes, making SLMAP a potential marker for treating lung adenocarcinoma [129]. STRIP2 was shown to promote NSCLC progression via interaction with insulin-like growth factor binding protein IGF2BP3 [83]. Expression of STRIP2 has also been shown to be positively correlated with phosphorylated (p) Akt, p-mTOR, and N-cadherin, while being negatively associated with protein levels of E-cadherin in lung adenocarcinoma cells [104]. These signalling proteins cooperate with STRIP2 in regulating tumourigenesis and aggressiveness of lung cancer cells. Research further highlighted STRIP2 in drug resistance in lung adenocarcinoma via regulating cellular immune responses [83]. 

### 6.8. Liver Cancer

Clinically, STRN is a promising diagnostic and prognostic indicator. Experimental lab work has also implicated STRN1 in hepatocarcinogenesis, regulating EMT by interacting with EMT markers. A negative expression correlation was identified between STRN1 and E-cadherin, contrasting with the expression of vimentin, an EMT marker known to maintain cellular integrity and increase cell susceptibility to stress during cancer progression. Vimentin was suppressed upon STRN knockdown [111]. Furthermore, the interaction between YSK1 and STRN1 was shown to facilitate EMT in HCC, regulating energy reserves through modifying cancer cells’ lipid metabolism [114]. The role of miRNA in cancer is also emerging, with STRN1 found to be a downstream target of miRNAs such as miR-199B-5P, which inhibits HCC progression by limiting the expression of STRN1 [130].

### 6.9. Colorectal Cancer

In colorectal cancer (CRC), knocking down STRN4 was shown to reduce the proliferation of colorectal cancer cells by modulating both cell cycle progression and apoptosis [86]. YSK1 was found to directly interact with the oncogenic protein LIM kinase (LIMK1), enhancing the invasiveness and malignancy of colon cancer cells by regulating the EMT process [94]. A higher level of MicroRNA-873, identified as a good prognostic indicator in CRC, restrains STRN4 expression and thereby inhibits CRC progression [131].

Despite the development of drugs and therapies, CRC remains the top leading cause of cancer death worldwide, partially attributed to the high prevalence of drug resistance of metastasized CRC patients. EGFR-targeted therapy is one strategy for tackling advanced CRC, and a recent study has reported a positive-feedback loop between YAP1 activation and EGFR/KRAS signalling. Therefore, targeting the STRIPAK complex to suppress YAP1 activation may shed light on resolving resistance to EGFR inhibitors [132].

### 6.10. Renal Cancer

STRIPAK genes have been documented to have strong prognostic value in kidney renal clear cell carcinoma (KIRC), yet the underlying mechanisms are still unclear [113]. However, several signalling pathways monitored by STRNs are known to take part in the regulation of renal cancer development [133,134]. As mentioned earlier, a number of the STRIPAK members can regulate YAP activation and translocation via the modulation of MST1/2 phosphorylation. Knocking down YAP reduces cell growth and induces cell apoptosis [134]. 

### 6.11. Head and Neck

In the context of head and neck squamous cell carcinoma (HNSCC), although MAP4K2 is not considered part of the STRIPAK complex, its close association with the complex members is crucial to maintaining tissue homeostasis in HNSCC, via regulation of YAP/TAZ [77]. Interestingly, the aberrant hyperactivation of YAP/TAZ, frequently observed in head and neck squamous cell carcinoma, provides further insight into targeting the STRIPAK complex in human papillomavirus (HPV) infection. This is particularly relevant, considering that the oropharynx, among the head and neck regions, is the site most frequently affected by HPV, exhibiting high YAP/TAZ activity [135].

### 6.12. Cervical Cancer

Exploration of STRIPAK in cervical cancer has recently emerged, with a study proposing signalling between the never in mitosis gene A (NIMA)-related kinase 2 (NEK2) protein, working in parallel with the STRIPAK complex to dephosphorylate MST1/2 and activate YAP in the Hippo signalling pathway [136]. Inhibition of MAP4K4 has been demonstrated to induce autophagy and increase the chemosensitivity of cervical cells to platinum-based therapy [137]. Similar to a few other cancer types, RNA interference of MAP4K4 also showed a promising effect in alleviating the invasiveness of cervical cancer cells [138]. 

### 6.13. Prostate Cancer

The extent to which STRIPAK complex members contribute to the development of prostate cancer remains largely unexplored. Previous research has unveiled the functional involvement of MST4 in prostate cancer through in vitro proliferation assays and in vivo observations. Overexpression of MST4 increases cell growth and tumour colonisation, whereas cells with reduced expression of MST4 exhibit suppressed tumourigenic behaviour [139]. The regulation by MST4 can be stimulated by EGFR agonists and may involve MAPKs signalling [140]. Additionally, STRIP1 and STRIP2 were shown to regulate the cytoskeletal dynamics of prostate carcinoma cells, albeit exhibiting only a mild restrictive effect on cells’ migration abilities in vitro. Cells with depleted STRIP1 or STRIP2 exhibit distinct phenotypes [73]. As part of the STRIPAK complex, it is possible that these two molecules, in conjunction with other complex members, are involved in other cytoskeleton-associated biological functions of cells, such as cytokinesis, and their dysregulation may contribute to prostate cancer development [73].

### 6.14. Bladder Cancer

In bladder cancer development, CCM3 was identified as a downstream target of anti-tumourigenic miR-26a-5p and miR-26b-5p. Both miRs were dysregulated in bladder cancer, leading to the upregulation of CCM3, which promotes proliferation of bladder cancer cells in vitro [103] CCM3 was further reported to have clinical significance [45,103], similar to STRN4, which was found to have elevated expression in bladder cancer tissue compared to adjacent normal tissue and was significantly associated with several clinicopathological factors of patients [102].

### 6.15. Other Cancer Types and Cancer-Related Incidences

Downregulation of the *CTTNBP2NL* gene was observed in oral squamous cell carcinoma [120,121]. In addition to the prognostic value of CCM3 in osteosarcoma, overexpression of CCM3 decreased cell susceptibility to apoptosis and stimulated tumourigenic phenotypes by activating the EMT pathway, whereas cells with reduced levels of CCM3 exhibited the opposite effects [93]. 

The cellular functions of STRIPAK members in peritoneal carcinomatosis (PC) have not been explicitly studied. However, a recent study reported the attenuation of PC upon YAP1 inhibition [141], whose activity has been closely associated with STRIPAK regulation. Another recent study demonstrated the interaction of STRIP1/2 with CCM3 in endothelial cells, collectively regulating blood-brain barrier integrity, which is crucial for cancer-induced brain metastasis [38]. 

## 7. Inhibitors Targeting STRIPAK Constituents

To date, there are no clinically proven drugs designed to disrupt the STRIPAK complex. However, studies have aimed to target and inhibit the core component of STRIPAK by designing small molecule inhibitors or peptides to assess the therapeutic potential of STRIPAK members. 

One such inhibitor, LB100, which targets PP2A, has been shown to have promising effects on enhancing chemotherapeutic potency in bladder cancer cells. It achieves this by inducing PP2A/p21 axis-dependent DNA damage and apoptosis [142,143]. Similar therapeutic-sensitiser effects have been reported in other cancer types such as small cell lung cancer and osteosarcoma [144,145]. Additionally, the STRN3-derived Hippo-activating peptide (SHAP), designed to target the PP2Aa–STRN3 interaction region, has demonstrated the restoration of Hippo activity and reduced gastric cancer tumour size in xenograft mouse models [79]. 

Combinational use of PARPi with other targeted therapies is emerging as a promising treatment in cancer [146]. This approach simultaneously stimulates several DNA repair-related lethal pathways in cancer cells. Recent research has found that inhibiting MST1/2 with its inhibitor XUM-MP-1 disrupts tumour-suppressive Hippo activity. This consequently facilitates tumour cells’ DNA repair, leading to resistance to oxaliplatin and PARPi in gastric HCG27 cells [80]. The cells’ sensitivity to treatment can be restored upon application of SHAP. 

Moreover, considering that SIKE1 and STRIP1 mediate recruitment of MST1/2 to the STRIPAK complex, two peptides (SAIP-1 and SAIP2) were further designed to disrupt interaction between STRN3–SIKE1 and STRN3–STRIP1 [80]. This disruption resulted in increased phosphorylation of MST1/2 and suppressed Hippo activity. The two peptides significantly elevated lethality to gastric cancer cells when used alongside PARPi treatment [80].

## 8. Conclusions

STRNs and other constitutive members of the STRIPAK complex are intriguing molecules to explore in the context of diagnosis, progression, and prognosis. As we have highlighted, there are links between STRIPAK complex members and the clinical outcome of patients, and a growing body of research in the past decade has postulated their roles in cancer and has evidently demonstrated their involvement in altering cell behaviour in a wide variety of cancer types. 

However, given the complex nature of the STRIPAK complex and the constant discovery of new STRIPAK complex members, some, like the kinases and phosphatase, show contrasting roles in regulating cell function. Consequently, inconsistent results are observed across cancer types and cellular events. Additionally, the constitution of the STRIPAK complex may vary in response to different cellular events, cell properties, or environmental stimuli, such as cell stress or variations in cell receptor status. Different forms of the STRIPAK complex give rise to various interactions that initiate and interfere with a variety of signalling cascades. Therefore, more research is needed to seek reproducibility and specific mechanisms corresponding to each of those changes in cell behaviour, during cancer development and progression.

## Figures and Tables

**Table 1:** Clinical significance of the STRIPAK complex members in different cancer types.

Cancer Type	STRIPAK Complex Members	Methods and Case Number	Expression Pattern in Cancer	Clinical Value	References
Oesophageal carcinoma	STRN4	Cohort study using serum samples (*n* = 192)	Upregulation	Poor prognostic indicator	[91]
	CCM3	Online database	Upregulation	Poor prognostic indicator	[45]
	STRN3	Online database (*n* = 40)	Upregulation	Positively associated with TNM stage, lymph node metastasis and pathological differentiation	[92]
Osteosarcoma	CCM3	Online database (*n* = 117) and IHC (*n* = 38)	Upregulation	Poor prognostic indicator	[93]
Colorectal cancer	YSK1	Online database (*n* = 230)	Upregulation	Higher expression associated with poor patients survival, TNM stage, and metastasis	[94]
Gastric cancer	STRN3	Cohort study (*n* = 88)	Upregulation	Positively correlated with tumour size, stage, lymphatic invasion, and lymph node metastasis; poor prognostic indicator	[79]
	STRN4	IHC (*n* = 30) and multiple online databases; cohort study using serum samples (*n* = 96)	Upregulation	Poor prognostic indicator	[91,95]
	MST3	IHC (*n* = 103)	Upregulation	Poor prognostic indicator	[96]
	MST4	Online database (n = 449)	Upregulation	Poor prognostic indicator; associated with lymph node metastasis, vascular invasion	[97]
	TRAF3IP3	Online database (*n* = 387)	Upregulation	Positively associated with tumour and lymph node stages	[98]
	CCM3	Online database	Upregulated		[45]
Brain lower grade glioma	CCM3	Online database	Upregulation	Poor prognostic indicator	[45]
Breast cancer	STRIP2	Cohort study (*n* = 127)	Upregulation	Higher in higher tumour grades; poor prognostic indicator	[87]
	STRN3	Multiple online databases and human biospecimens	Upregulation	Higher in tumour tissues; negatively associated with tumour progression; expression associated with cancer subtypes	[8,99]
	CCM3	Cohort study (*n* = 127); online database (*n* = 1809)	Upregulation	Poor prognostic indicator, expression is subtype dependent and associated with lymph node metastasis	[87,99]
	MINK1	Cohort study (*n* = 127)	Upregulation	Poor prognostic indicator	[87,99]
	SLMAP	Cohort study (*n* = 127)	Upregulation	Poor prognostic indicator	[87]
	MOB4	Cohort study (*n* = 127)	Upregulation	Poor prognostic indicator	[87]
	STRN4	Cohort study (*n* = 127); online database (*n* = 1809); cohort study using serum samples (*n* = 96)	Upregulation	Poor prognostic indicator, expression associated with cancer subtypes	[87,91,99]
	STRN1	Cohort Study (*n* = 127)	Upregulation	Higher in tumour	[87]
	MST3	Immunoblotting (*n* = 20) and multiple online database	Upregulation	Upregulated in tumour and TNBC subtype of breast cancer patients; poor prognostic indicator	[100]
	MST4	Multiple online databases and IHC (93); online database (*n* = 1089)	Upregulation	Associated with lymph node metastasis; poor prognostic indicator	[99,101]
Bladder cancer	STRN4	IHC (*n* = 28)	Upregulation	Poor prognostic indicator, positively associated with tumour size, muscle invasion depth, tumour grade	[102]
	CCM3	Online databases	Upregulation	Poor prognostic indicator	[45,103]
Non-small cell lung cancer	STRIP2	IHC (*n* = 189)	Upregulation	Poor prognostic indicator; higher in tumour tissues	[83]
	STRIP2	Clinical cohort (*n* = 51), IHC (*n* = 189) and multiple online databases	Upregulation	Poor prognostic indicator; associated with poor tumour differentiation, TNM stage, lymph node metastasis, and cancer thrombus	[83]
Lung adenocarcinoma	STRIP2	Multiple online databases	Upregulation	Poor prognostic indicator; negatively associated with immune response	[83,104,105]
	CCM3	Online database	Upregulation	Good prognostic indicator, positively correlated with expression of tumour-infiltrating cells	[45]
	MST3	Online database (*n* = 573)	Upregulation	Poor prognostic indicator	[106]
	TRAF3IP3	Multiple online databases	Downregulation	Good prognostic indicator	[107]
	STRN4	Cohort study using serum samples (*n* = 96)	Upregulation	Poor prognostic indicator	[91]
Pituitary adenomas	CCM3	Online database (*n* = 14)	Upregulation	Poor prognostic indicator	[64]
Pancreatic cancer	CCM3	Multiple online databases	Upregulation	Higher expression associated with unfavourable outcome	[45]
	MAP4K4	Multiple datasets and IHC (*n* = 52; *n* = 66)	Upregulation	Positively associated with tumour stage; poor prognostic indicator, associated with metastasis, tumour size, and lymph node positivity	[84,108]
	PPP2R1B (PP2A subunit A Beta isoform)	Multiple online databases	Downregulation	Good prognostic indicator	[109]
	STRN1	Multiple online databases	Upregulation	Poor prognostic indicator	[109]
	STRN3	Multiple online databases	Upregulation	Poor prognostic indicator	[109]
Prostate cancer	CCM3	IHC (*n* = 160)	Upregulation	Positively associated with age	[110]
	MST4	IHC (*n* = 160)	Upregulation	Positively associated with age	[110]
	YSK1	IHC (*n* = 160)	Upregulation	Higher expression is tumour with grades 3–5 compared with grade 1–2	[110]
Liver hepatocellular carcinoma	STRN	IHC (*n* = 45)	Upregulation	Positively correlated with TNM stage and lymph node metastasis	[111]
	STRN4	Clinical cohort (*n* = 521)	Upregulation	Poor prognostic indicator	[112]
	PP2AA	Online database (529)	Upregulation	Poor prognostic indicator; associated with tumour grade, stage	[113]
	YSK1	IHC (*n* = 29) and online database (*n* = 365)	Upregulation	Poor prognostic indicator, associated with TNM, vascular invasion	[114]
	CCM3	Online database	Upregulation	Poor prognostic indicator	[45]
	MST4	Clinical cohort (*n* = 178) and IHC (*n* = 178)	Upregulation	Positively associated with tumour size, vascular invasion, metastasis, TNM stages; poor prognostic indicator	[115]
	MST4	IHC (*n* = 325)	Downregulation	Tumour-suppressive, negatively associated with T stage, nodal status, and metastasis; good prognostic indicator	[116]
	TRAF3IP3	Online database (*n* = 303)	Downregulation	Good prognostic indicator; positively correlated with immune-checkpoint gene *PDCD1*	[117]
Head and Neck	CCM3	Online database	Upregulation	Poor prognostic indicator	[45]
Kidney renal clear cell carcinoma	TRAF3IP3	Online database (*n* = 623)	Upregulation	Poor prognostic indicator	[113]
Kidney chromophobe carcinoma	CCM3	Online database	Upregulation	Poor prognostic indicator	[45]
Osteosarcoma	CCM3	Clinical cohort (*n* = 38)	Upregulation	Poor prognostic indicator for five-year mortality	[93]
Glioma	TRAF3IP3	Multiple online databases	Upregulation	Poor prognostic indicator; associated with grade, EGFR mutation, and worse histologic type	[118,119]
Uterine corpus endometrial carcinoma	CCM3	Online database	Upregulation	Poor prognostic indicator	[45]
Oral squamous cell carcinoma	CCM3	Online database	Upregulation	Poor prognostic indicator	[45]
	CTTNBP2	Multiple online databases	Downregulation		[120,121]

## Abbreviations

AKT	AKT Serine/Threonine Kinase 1
ALK	Anaplastic Lymphoma Kinase
APC	Adenomatosis Polyposis Coli Tumour Suppressor
CCM3	Cerebral Cavernous Malformations 3; or PDCD10/TFAR15
CDK	Cyclin-Dependent Kinase
CK1	Casein kinase 1
CTTNBP2	Cortactin binding protein 2,
EMT	Endothelial-to Mesenchymal Transition
ERM	Ezrin, Radixin and Moesin protein family
FAM40	Family With Sequence Similarity 40
*FGFR1OP2*	Fibroblast Growth Factor Receptor 1 Oncogene Partner 2,
FZD	Frizzled Class Receptor
GCKs	Germinal centre kinases
GSK3	Glycogen synthase kinase 3,
HNSCC	Head and Neck Squamous Cell Carcinoma
IGFBP3	Insulin-Like Growth Factor 2 MRNA Binding Protein 3
LEF1	Lymphoid Enhancer Binding Factor
LATS1/2	Large tumour suppressor kinase
MAP4Ks	Mitogen-Activated Protein Kinase Kinase Kinase Kinases
MINK1	Misshapen-like kinase 1, or MAP4K6 (Mitogen-Activated Protein Kinase Kinase Kinase Kinase 6)
MOB4	Monopolar spindle one binder family member 4
MST1	Mammalian sterile20-like kinases or Ysk1
NF-κB	Nuclear Factor Of Kappa Light Polypeptide Gene Enhancer In B Cells 1
NSCLC	Non-Small Cell Lung Cancer
PDCD10	Programmed Cell Death 10, or CCM3/TFAR15
PI3K	Phosphatidylinositol-4,5-Bisphosphate 3-Kinase or Phosphoinositide-3-Kinase
PP2A	Protein Phosphatase 2 Phosphatase Activator, or Serine/(PPP2R4)Threonine-Protein Phosphatase 2A Regulatory Subunit 4
PP2AC	Serine/Threonine-Protein Phosphatase 2A Catalytic Subunit, or PPP2CA (Protein Phosphatase 2 Catalytic Subunit Alpha)
RAS	RAS proto-oncogene
SAV1	Salvador Family WW-Domain-Containing Protein 1 or Salvador homolog 1,
SG2NA	Cell Cycle Autoantigen SG2NA, also known as Striatin–3
SIKE1	Suppressor of IKK epsilon-1
SLIK	Ste20-like kinase, or KIAA0204
SLMAP	Sarcolemmal membrane-associated protein, also known as KIAA1601
STRIP1	striatin interactive protein-1, also known as Family With Sequence Similarity 40, Member A, FAM40A, or KIAA1761
STRIP2	striatin interactive protein-2, also known as Family With Sequence Similarity 40, Member B, FAM40B, or KIAA1761
STRIP2	striatin interactive protein-2, also known as KIAA1170
STRIPAK	Striatin-interacting phosphatase and kinase
STRN	Striatin
TAZ	WW-Domain-Containing Transcription Regulator 1 or Transcriptional Coactivator with PDZ-Binding Motif
TCF1	T-cell factor 1
TEAD1	TEA Domain Family Member 1 (SV40 Transcriptional Enhancer Factor)1, or Transcriptional Enhancer Factor TEF-1, or TEF1
TFAR15	TF-1 Cell Apoptosis-Related Protein 15, CCM3/PDCD10
TNIK	TRAF2 And NCK-Interacting Protein Kinase
TRAF3	TNF Receptor Associated Factor 3
TRAF3IP3	TRAF3-interacting protein 3
VEGF	Vascular-endothelial growth factor
WNT	Wingless-Type MMTV Integration Site Family
YSK1	Sterile 20/Oxidant Stress-Response Kinase 1, or Serine/Threonine Kinase 25 (STK25)
